# NF-κB Mutations in Germinal Center B-Cell Lymphomas: Relation to NF-κB Function in Normal B Cells

**DOI:** 10.3390/biomedicines10102450

**Published:** 2022-10-01

**Authors:** Laura Pasqualucci, Ulf Klein

**Affiliations:** 1 Institute for Cancer Genetics, Department of Pathology & Cell Biology, The Herbert Irving Comprehensive Cancer Center, Columbia University, New York, NY 10032, USA; 2 Division of Haematology & Immunology, Leeds Institute of Medical Research at St. James’s, University of Leeds, Leeds LS9 7TF, UK

**Keywords:** lymphoma, lymphomagenesis, B cell, B-cell development, germinal center, NF-κB signaling pathway, genetic aberration, diffuse large B-cell lymphoma, Hodgkin lymphoma

## Abstract

Most B cell lymphomas arise from the oncogenic transformation of B cells that have undergone the germinal center (GC) reaction of the T cell-dependent immune response, where high-affinity memory B cells and plasma cells are generated. The high proliferation of GC B cells coupled with occasional errors in the DNA-modifying processes of somatic hypermutation and class switch recombination put the cell at a risk to obtain transforming genetic aberrations, which may activate proto-oncogenes or inactivate tumour suppressor genes. Several subtypes of GC lymphomas harbor genetic mutations leading to constitutive, aberrant activation of the nuclear factor-κB (NF-κB) signaling pathway. In normal B cells, NF-κB has crucial biological roles in development and physiology. GC lymphomas highjack these activities to promote tumour-cell growth and survival. It has become increasingly clear that the separate canonical and non-canonical routes of the NF-κB pathway and the five downstream NF-κB transcription factors have distinct functions in the successive stages of GC B-cell development. These findings may have direct implications for understanding how aberrant NF-κB activation promotes the genesis of various GC lymphomas corresponding to the developmentally distinct GC B-cell subsets. The knowledge arising from these studies may be explored for the development of precision medicine approaches aimed at more effective treatments of the corresponding tumours with specific NF-κB inhibitors, thus reducing systemic toxicity. We here provide an overview on the patterns of genetic NF-κB mutations encountered in the various GC lymphomas and discuss the consequences of aberrant NF-κB activation in those malignancies as related to the biology of NF-κB in their putative normal cellular counterparts.

## 1. Introduction

B-cell lymphomas comprise a heterogeneous group of tumours that, in most cases, originate from the malignant transformation of cells in the germinal center (GC). A potential oncogenic role for the NF-κB signaling pathway in lymphomas was originally postulated few years after the discovery of the pathway in the 1980s, based on the observation of constitutive NF-κB activity in classical Hodgkin lymphoma (cHL) cell lines [1]. This finding could be explained in part by the frequent association of cHL with latent infection by the Epstein–Barr Virus (EBV), as the *LMP1* and *LMP2A* genes encoded by EBV had been shown to mimic signals associated with engagement of the CD40 and B-cell receptor (BCR), respectively, which stimulate the NF-κB signaling pathway [1]. However, NF-κB activity was also detected independently of EBV infection, suggesting the existence of additional underlying mechanisms and prompting the search for genetic lesions in constituents of the pathway. Moreover, two studies in 1991 reported the presence of structural alterations in two components of the NF-κB signaling pathway, which can lead to constitutive and possibly oncogenic NF-κB activation in B-cell cancers. In the first study, a gene homologous to the REL transcription factors, later designated as *NFKB2*, was cloned from a translocation breakpoint in a diffuse large B-cell lymphoma (DLBCL) case [2]. The second study reported the discovery of rearrangements or amplifications of the *REL* locus (encoding the NF-κB subunit c-REL) in two follicular lymphoma cases and one DLBCL [3]. Subsequent studies published between 1999 and 2000 then provided a definitive genetic link between NF-κB and cHL, by identifying loss-of-function mutations in the gene encoding for a negative regulator of this pathway, IκBα [4,5,6]. Of note, inactivating mutations of IκBα occurred predominantly in EBV-negative cHL cases, indicating that NF-κB is aberrantly activated through different mechanisms in cancer, and that EBV infection can substitute for some genetic lesions in cHL cells. Since then, several studies have documented the involvement of NF-κB in other types of lymphoma. In 2001, following the phenotypic distinction of DLBCL into subtypes that arise from B-cells at different stages of normal differentiation [7], it was found that cell lines corresponding to the activated B cell-like DLBCL (ABC-DLBCL) subtype display constitutive activation of the NF-κB pathway, although the underlying mechanism remained elusive [8]. Advances in sequencing technologies and loss-of-function RNA interference screens conducted between 2007 and 2011 then established aberrant NF-κB pathway activation as a frequent event in B-lineage malignancies that is sustained by somatic genetic mutations and contributes to their pathogenesis. In particular, several studies identified NF-κB pathway mutations in multiple myeloma (MM) and ABC-DLBCL [9,10,11,12,13,14,15,16,17], as well as in other lymphoma subtypes not covered in this review.

At the same time, advances in the phenotypic and histologic characterization of normal GC B-cell subpopulations, together with new insights into the molecular control of GC and post-GC B-cell development, established a framework for more accurately assigning B-lineage malignancies to their putative normal cellular counterparts [18,19,20,21,22,23,24,25,26,27]. What emerged from these studies is that B-cell lymphomas often highjack genes and/or biological pathways with critical functions in the development and physiology of GC B cells by a variety of genetic alterations [26,28,29,30,31,32]. In this review, we discuss how genetic alterations in NF-κB pathway components may functionally contribute to the transformation of B cells undergoing the GC reaction, focusing on DLBCL, cHL, and primary mediastinal large B-cell lymphoma (PMBL).

## 2. NF-κB Signaling Pathway and Transcription Factor Subunits

**Figure 1:**
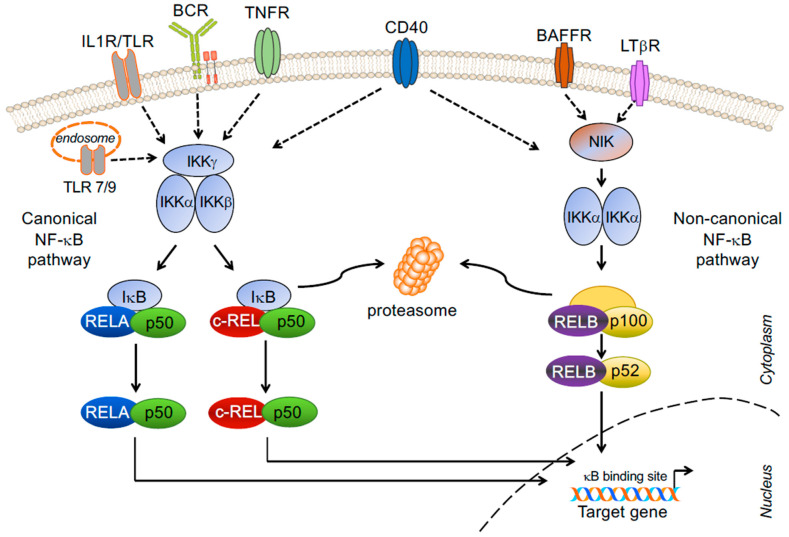
NF-κB subunits and signaling pathways in B cells. Stimulation via cell surface receptors leads to activation of the IKK complex or NIK, the upstream regulators of the canonical and non-canonical NF-κB pathway, respectively (adaptor molecules not shown). In the canonical pathway, phosphorylation of IκB by IKKs causes its proteasomal degradation, thus releasing the NF-κB dimers RELA/p50 and c-REL/p50 for nuclear translocation. In the non-canonical pathway, NIK activates IKKα, which in turn induces the processing of the p100 inhibitor κB subunit to p52, thus releasing the NF-κB dimer RELB/p52 for nuclear translocation. Only RELA, c-REL and RELB contain transactivation domains that drive transcription of NF-κB target genes.

Thirty-six years ago, Ranjan Sen in the Baltimore laboratory named a protein bound to the immunoglobulin light chain enhancer ‘nuclear factor-κB’ [33]. This observation rapidly led to the discovery of a major signal transduction pathway with critical roles in innate and adaptive immunity [34], although its activity is not confined to the immune system. NF-κB signaling plays fundamental roles in a multitude of biological processes; broadly speaking, NF-κB regulates the expression of genes involved in cell growth and survival, inflammation and stress responses [35,36,37]. Mechanistically, signals emanating from various surface receptors are ultimately transmitted to five transcription factor subunits, representing the downstream mediators of the pathway, which can occur as heterodimers or homodimers: RELA (p65), RELB, c-REL, p50 (and the precursor p105, or NF-κB1) and p52 (and the precursor p100, or NF-κB2). Only RELA, RELB and c-REL contain transactivation domains at their C-terminus that drive transcription. In resting cells, NF-κB heterodimers or homodimers are sequestered in the cytoplasm by inhibitory NF-κB proteins. Upon engagement of cell surface receptors, the activation of a signaling cascade culminates in the release of the subunits and their translocation to the nucleus, where they bind to specific sequences regulating transcription of NF-κB target genes. Two separate NF-κB pathways, the canonical and the non-canonical pathway, integrate the biological functions instructed by the activation of different cell surface receptors [Figure 1].

RELA, c-REL and p50 are the subunits of the canonical (or classical) NF-κB pathway that in B cells is activated by the BCR, Toll-like receptors (TLRs), and the members of the tumour necrosis factor receptor (TNFR) super family CD40, CD30 and Receptor Activator of Nuclear factor-κB (RANK/TNFRSF11A) [38,39] [Figure 1]. These receptors utilize multiple adaptor proteins to relay signals from the microenvironment to the major heterodimers of this pathway, RELA/p50 and c-REL/p50, which in resting cells are sequestered in the cytoplasm by the Inhibitory κB proteins IκBα, IκBβ and IκBε. Upon receptor activation, phosphorylation of IκBα by the IκB kinase (IKK) complex, composed of two catalytic subunits (IKKα and IKKβ) and the regulatory subunit IKKγ (also known as NEMO), marks the IκB protein for proteasome-mediated degradation, thus releasing the canonical NF-κB heterodimers for nuclear translocation. Important negative regulators of the canonical pathway are the deubiquitinating enzymes A20, encoded by *TNFAIP3*, and CYLD, which act upstream of the IKK complex.

RELB and p52 are the subunits of the non-canonical (or non-classical or alternative) NF-κB pathway, which can be activated in B cells by CD40 (via the CD40 receptor), B-cell activating factor (BAFF) (through the BAFF receptor), and/or lymphotoxin-β (LTβ) (via the LTβ receptor) [38,39]. In normal conditions, the non-canonical pathway is maintained inactive through the rapid turn-over of NF-κB-inducing kinase (NIK), which is trapped in a multiprotein inhibitory E3 ubiquitin ligase complex composed of TRAF2, TRAF3, and cIAP1/2. In particular, TRAF3 has been identified as a major negative regulator of NIK, which constantly induces its degradation by the proteasome [37]. Following receptor stimulation, the release of NIK from this multi-subunit complex activates the IKKα homodimer and induces the processing of p100 (bound to RELB) to p52, allowing for the nuclear translocation of RELB/p52 heterodimers; thus, p100 is the inhibitory ĸB protein of the non-canonical NF-κB pathway. Of note, stimulation through CD40 has been shown to activate both pathways. In addition, evidence suggests a crosstalk between the two pathways in some contexts [40,41]. Thus, NIK can also activate the canonical pathway, and canonical NF-κB subunits can upregulate the transcription of the non-canonical NF-κB subunits (although this will not lead to an activation of the non-canonical pathway). Other kinases activating NF-κB include the unconventional IKK family members IKK1 and TBK1, although the underlying mechanisms remain unclear. The potential crosstalk between the canonical and non-canonical NF-κB pathways needs to be taken into account when approaching the design of strategies aimed at inhibiting NF-κB by targeting the separate pathways.

## 3. Role of NF-κB Signaling Pathways and Subunits in GC B-Cell Development

### 3.1. The Germinal Center B-Cell Reaction

**Figure 2:**
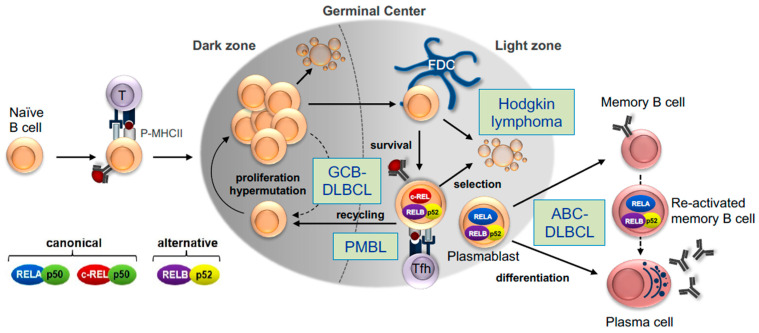
Germinal center B-cell development, NF-κB transcription factors and putative cellular derivation of GC lymphomas. Schematics of the major GC B-cell developmental stages, with critical NF-κB transcription factor subunits involved in the reaction. The memory B-cell re-activation step is also shown. Boxes indicate the putative relation of GC-derived B-cell lymphomas to their normal cellular counterparts. NF-κB c-REL and the RELB/p52 heterodimer are independently required for the recirculation of light zone B cells to the dark zone, i.e., the population that presumably gives rise to GCB-DLBCL, suggesting that these transcription factors may play a role in the pathogenesis of GCB-DLBCL and potentially PMBL. NF-κB RELA is required for plasmablastic development, which is associated with the ABC-DLBCL cell-of-origin, suggesting a possible role for RELA in ABC-DLBCL lymphomagenesis. cHL is thought to originate from the transformation of GC B cells with crippled Ig variable region genes that would normally apoptose. Tfh, T follicular helper cell. FDC, follicular dendritic cell; P-MHCII, peptide-MHC class II complex.

Over the past decade, advances in methodology and mouse modeling were instrumental in identifying and characterizing the developmental stages of antigen-activated B cells during the GC reaction, as well as the transcription factor networks that control this unique microenvironment. The GC reaction is initiated in the T cell-dependent immune response upon pathogen invasion and generates high-affinity memory B cells and plasma cells that clear the infection and provide long-lasting humoral immunity [23,42]. The GC reaction is essential for the body’s defense against infections, as documented by the severity of primary immunodeficiency syndromes in which this response is functionally impaired due to inherited germline mutations in critical regulators of the reaction [43]. Guided by T-cell help, antigen-activated GC precursor B cells enter the primary follicle, where they divide for several days at a very fast rate. A week after the initial antigen-encounter, a polarized GC microenvironment is established that comprises a histologically defined dark zone and light zone. During their proliferation, dark zone B cells (also referred to as centroblasts) diversify their rearranged immunoglobulin (Ig) variable region genes by the process of somatic hypermutation to generate a variety of antibody mutants [Figure 2]. These cells then differentiate into more quiescent light zone B cells (or centrocytes), where antibody mutants are positively selected for improved antigen affinity through interactions with the antigen, bound to follicular dendritic cells, and with T-follicular helper (Tfh) cells; alternatively, and in most cases, cells undergo apoptosis, because of inferior antigen binding or the acquisition of mutations that debilitate the antibody structure. Positively selected GC B cells go through iterative cycles of somatic hypermutation and selection between the dark and light zones in a process known as “cyclic re-entry” and eventually leave the GC as memory B cells or long-lived plasma cells. The GC is also the site where B cells undergo class switch recombination, a DNA recombination process that confers distinct effector functions to antibodies with the same specificity, although recent evidence suggests that this event occurs predominantly after B-cell activation and before the B cells enter the follicle [44].

The three developmental stages of the GC reaction –initiation, maintenance (including the selection stage) and differentiation into memory B cells or long-lived plasma cells– are the subject of transcriptional, post-transcriptional and epigenetic control mechanisms [19,23,25,27,31,45]. Over the past decades, our understanding of the molecular mechanisms underlying these phases and their relationship with the malignant transformation process has progressed significantly through synergistic research efforts in the areas of B-cell immunology and lymphomagenesis. Thus, the identification and molecular characterization of B-cell subsets corresponding to distinct cell stages in development has provided new insights into the cellular derivation of B cell lymphomas and the underlying pathogenic mechanisms. Conversely, the function of many key B cell-developmental regulators has been revealed only after their genes were cloned from recurrent genetic lesions in lymphomas (e.g., *BCL2*, *BCL6*, *MYC*). Indeed, a common theme that emerged from these studies was that the genetics-driven alteration of pathways normally involved in B-cell development represents a major contributor to the malignant transformation of B cells [26,28,29,30,31,32].

### 3.2. NF-κB Signaling in the GC Reaction

A fundamental role for NF-κB signaling in the GC reaction was always expected: the stimulation of CD40 on B cells by CD154 expressed on T helper cells activates NF-κB, and inherited germline mutations in the *CD40* and *CD154* genes that impair CD40-CD154 co-stimulation cause the Hyper-IgM syndrome, in which GCs are absent [43]. Moreover, NF-κB activation has long been known to be required for cell growth in in vitro-activated lymphocytes [38,39]. On this background, it was rather surprising to learn, in the early 2000s, that the vast majority of GC B cells do not show active NF-κB signaling, as determined by the lack of a NF-κB target gene signature in two independent DNA microarray analyses [46,47]. In accordance, immunohistochemical analysis of tonsillar tissue sections demonstrated the absence of nuclear NF-κB transcription factors in the vast majority of GC dark zone and light zone B cells [46], further validating the inactive state of the pathway. However, a subset of GC B cells in the light zone did show nuclear translocation of both canonical [46] and non-canonical [48] NF-κB subunits. Although it is not known whether these subunits accumulate in the nucleus of the same cell or of different cells, the identification of nuclear NF-κB^+^ light zone B cells pointed towards a functional role for NF-κB transcription factors in discrete phases of GC B-cell development. The requirement for NF-κB signaling in B cells at the initial antigen–activation step had long been established from the study of NF-κB-subunit constitutional knockout mice [38,39]. More recently, studies with conditional alleles allowing the deletion of individual NF-κB subunits in GC B cells provided news insights into the function of NF-κB activation during the GC reaction [25]. These studies revealed that individual NF-κB transcription factors control distinct biological programs that are required at specific stages during GC B-cell differentiation, including antigen-activation, GC maintenance and plasma cell differentiation.

### 3.3. Canonical Subunit c-REL

The canonical NF-κB subunit c-REL is required for GC formation upon T cell-dependent immunization [49,50,51] in a B cell-intrinsic fashion [52]. This requirement was demonstrated through in vitro-stimulation experiments with several different mitogens in B cells carrying a conditional *Rel* (encoding c-REL) allele. In this mouse model, when Cre expression was upregulated two days after the initial antigen-activation, GCs formed apparently normally but started to gradually involute after day 7 [53], which is the time after immunization when polarized GCs consisting of dark zone-light zone have fully formed and selection for high-affinity antibody mutants begins. By day 14, GCs almost completely disappeared, following the loss of both dark zone and light zone B cells. A similar phenotype had previously been shown upon ablation of MYC function in the GC [54,55], suggesting that, in analogy to those studies, c-REL is required for the recirculation of positively selected light zone B cells back to the dark zone, and thus for the maintenance of the GC reaction. c-REL and MYC are likely to be active in the same light zone B cells, since the transcriptional profile of the Myc-positive light zone subset comprises an NF-κB signature [54,55]. This notion is further supported by the finding that, in GC B cells, CD40-mediated activation of NF-κB signaling by Tfh cells is required in parallel with BCR activation to induce MYC expression [56]. Functionally, evidence suggests that, in light zone B cells (and presumably also during the initial antigen-activation), c-REL controls the expression of a glycolytic metabolic program that generates energy and building blocks for cell growth [53], which in turn prepares the selected B cells to undergo cell division associated with re-entering the dark zone [57,58,59]. The extent to which c-REL and MYC crosstalk with each other within light zone B cells is unknown. Interestingly, using a mouse model in which c-REL is specifically overexpressed in B cells, it has recently been demonstrated that increased c-REL protein dosage causes an expansion of GC B cells [60], further highlighting the importance of this NF-κB subunit in GC B-cell physiology.

Finally, deficiency of c-REL in humans can be a cause for combined immunodeficiency (CID) [61,62]. The associated phenotype, which comprises lack of memory B cells, lack of isotype switched antibodies, and impaired B-cell proliferation, is consistent with the phenotype observed in the conditionally deleted *Rel* mice, suggesting that the observed human B-cell phenotype is cell autonomous.

### 3.4. Canonical Subunit NF-ĸB1

The inability to process p50 from its precursor p105 in p105-mutant mice, effectively preventing the generation of c-REL/p50 and RELA/p50 heterodimers, impaired the formation of GCs upon T cell-dependent immunization [63]. Since c-REL is critically required for the initial antigen-activation step, this finding suggests that the inability of the c-REL/p50 complex to translocate into the nucleus in p105-mutant mice prevents B-cell activation and further differentiation. Conversely, constitutional deletion of the *Nfkb1* gene (i.e., neither p50 nor its p105 precursor is produced) caused an increase in the formation of spontaneous GCs in aging mice compared to NF-κB1-proficient mice [64]. The observed phenotype in these mice of chronic disease pathogenesis may in part be explained by enhanced c-REL activity in *Nfkb1*-deficient B cells, since p105 is essentially an inhibitory ĸB protein for c-REL. The absence of p105 may shift the spectrum of NF-κB heterodimers and homodimers towards a higher amount of c-REL containing complexes that enter the nucleus upon canonical NF-κB pathway activation.

In humans, loss-of-function mutations of NF-κB1 represent the most common NF-κB subunit deficiency; the affected individuals present with common variable immunodeficiency (CVID) which leads to a somewhat milder immunodeficiency phenotype compared to the CID observed for c-REL deficiency, and is mostly accompanied by a reduction in isotype switched memory B cells [65,66,67]. This phenotype is at least partly consistent with reduced c-REL activity, which forms a heterodimer with p50.

### 3.5. Canonical Subunit RELA

RELA and c-REL are functionally redundant canonical NF-κB subunits in the B-cell developmental stages up to the generation of naïve B cells [52,68]. However, the functions of the subunits diverge at the antigen-activation step, where RELA, unlike c-REL, was found to be dispensable for the formation of GCs in a B-cell autonomous fashion [52]. When Cre was expressed in conditional *Rela* knockout mice after T-dependent B-cell activation, GC maintenance was not affected, unlike observed for c-REL [53]. However, RELA was required for the generation of long-lived plasma cells in the GC, presumably in part reflecting the inability to fully upregulate the expression of the plasma cell developmental regulator BLIMP1 [53]. These results were consistent with findings from computational analyses indicating that c-REL and RELA have differential roles in the developmental steps that ultimately lead to the generation of plasma cells [69]. Indeed, c-REL expression is strongly downregulated at both transcriptional and protein levels in human and murine plasma cells [48,70]. Overall, the available evidence suggests that RELA is the exclusive transcriptionally active canonical NF-κB subunit in GC-derived, long-lived plasma cells.

In humans, no primary immunodeficiencies linked to RELA defects have been identified until now. This is presumably for two reasons. First, haploinsufficiency of RELA, in contrast to c-REL, has no effect on B-cell development; RELA haploinsufficiency has been identified as the cause of chronic mucocutaneous ulceration, which however affects stromal cells [71]. Second, homozygous deletion of RELA in the mouse germline causes embryonic lethality [72], suggesting that homozygous loss-of-function mutations in humans are incompatible with life.

### 3.6. Non-Canonical Subunits RELB and NF-ĸB2

Functional ablation of the non-canonical NF-κB pathway, obtained by deleting the genes encoding the RELB and NF-κB2 transcription factors in GC B cells, caused an involution of the GC past day 7 after T cell-dependent immunization, mirroring the phenotype observed upon *Rel* deletion [48]. The non-redundant roles in this process of c-REL on the one hand and the non-canonical NF-κB pathway on the other are presumably the result of different biological programs controlled by the respective transcription factors. Evidence suggests that the RELB/p52 heterodimer is required in differentiating GC B cells for cell cycle progression and for the efficient production of proteins [48]. Compared to surrounding lymphocytes, the NF-κB2 protein is strongly expressed in plasma cell precursors within the GC and in plasma cells in human tonsils [48]. Interestingly, while the deletion of *Nfkb2* alone in the conditional mice did not significantly impair GC maintenance, the fraction of long-lived plasma cells was dramatically reduced compared to the NF-κB2-proficient controls, suggesting that this subunit is required for the development or physiology of plasma cells [48]. Since *Relb* deletion in the GC did not have any measurable effect on GC and plasma cell development, these findings suggest a role for p52 homodimers and/or p52/RELA heterodimers in the development of long-lived plasma cells, which remains to be elucidated.

In humans, RELB loss-of-function was found to give rise to CID similar to what was observed for c-REL deficiency [73]. Since, in mice, conditional homozygous *Relb* deletion in various B-cell subsets had no effect [48,74], the observed defects in the *RELB*-mutant patients may be due to a RELB-dependent impairment in the function of other cell types. The latter notion is supported by the phenotype of constitutional *Relb* knockout mice that show severe defects in lymphoid organization and do not form GCs [38]. An alternative explanation is that the function of RELB may differ between species. Loss-of-function mutations of NF-κB2 were the first mutations found in NF-κB subunits that have been associated with primary immune deficiency, leading to a reduction in switched memory B cells [75,76,77]. The phenotype observed upon conditionally deleting *Nfkb2* in murine GC B cells, specifically the absence of long-lived plasma cells, suggests that B-cell autonomous components contribute to the immune deficiency that results from defective lymphoid organ development. Of note, activating mutations have been recently identified in *NFKB2*, which lead to immunodeficiency in a different manner [78]. Thus, the investigation of the roles of distinct NF-κB subunits in primary immunodeficiency remains an area of active investigation that is far from over.

In summary, it is becoming increasingly clear that the individual NF-κB transcription factors exert unique functions during different stages of B-cell development. The possibility that NF-κB subunits have distinct roles also in the biology of a malignancy should be taken into consideration when determining the oncogenic effects of aberrant NF-κB activation in B-cell lymphomas, as this knowledge may be exploited for the development of more specific drugs with reduced systemic toxicity.

## 4. Dysregulation of the NF-κB Signaling Pathway in DLBCL

### 4.1. Cell of Origin and Classifications

DLBCL, the most common lymphoma diagnosed in adults, is a complex and aggressive disease comprising multiple molecularly, phenotypically, and clinically distinct entities [32,79]. A major advance in the understanding of this heterogeneity came from gene expression profile studies that led to the recognition of three molecular subgroups—ABC-DLBCL, GC B-cell type (GCB)-DLBCL, and unclassified DLBCL—based on the transcriptional similarities with B-cell populations at various stages of post-antigen encounter differentiation [7]. This first classification, commonly referred to as COO (cell-of-origin) and now officially incorporated into the updated WHO-classification, has been further refined through the integration of genomic data in two seminal studies that allowed the identification of as many as 7 genetic subgroups displaying distinct patterns of concurrent genetic alterations [80,81,82]. In the “LymphGen” taxonomy, the ABC-DLBCL is further divided into four genetic subtypes, named after their “seed” genetic alterations: MCD (*MYD88* and *CD79B^L265P^* mutations), N1 (*NOTCH1* mutations), BN2 (*BCL6* translocations and *NOTCH2* mutations), and A53 (aneuploidy and *TP53* genetic alterations). In contrast, GCB-DLBCL is predominantly enriched in EZB (*EZH2* mutations and *BCL2* translocations, either MYC translocation positive or negative) and ST2 (*SGK1* and *TET2* mutations) DLBCL, a smaller subset of cases being assigned to the BN2 and A53 subtype [80]. The non-negative matrix factorization (nNMF)-based classifier recognized 5 subgroups (C1-C5), with significant concordance between the so-called Cluster 5 (C5) and MCD-DLBCL, C3 and EZB-DLBCL, C1 and BN2-DLBCL, C2 and A53-DLBCL, and C4 and ST2-DLBCL [80,82]. These subgroups are associated with differential response to therapy, underscoring the potential clinical relevance of this new taxonomy that has been independently validated [83,84]. Nonetheless, discrepancies remain between the two classifiers in the way cases are assigned to each group, and a significant fraction of tumours cannot be assigned to a definitive subtype by the LymphGen algorithm. Moreover, COO gene expression groups maintain significantly disparate outcomes within BN2 and A53 subtypes, emphasizing the need to consider both genetic and phenotypic characteristics when interpreting the impact of precision-medicine approaches.

**Figure 3:**
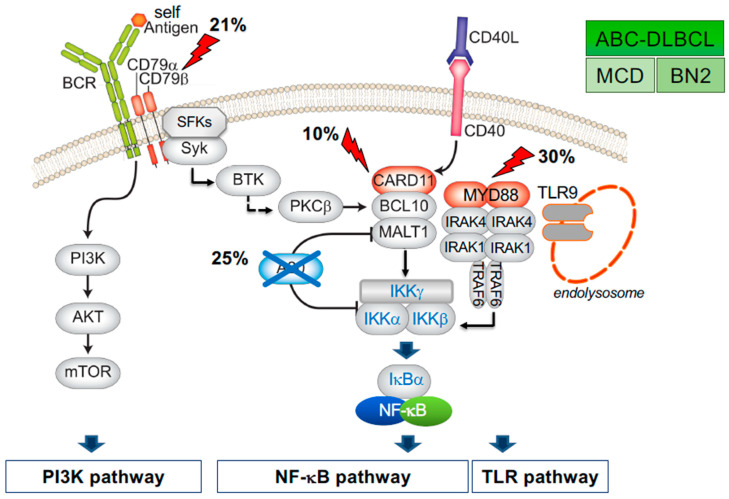
BCR-dependent constitutive activation of the canonical NF-κB signaling pathway in ABC-DLBCL. Genetic aberrations enforcing constitutive activation of the NF-κB signaling cascade downstream of the BCR, CD40 receptor, and TLR9 are a genetic hallmark of ABC (MCD/C5 and BN2) DLBCL. Direct targets of genetic lesions are shown in shades of blue (inactivation) and red (deregulated expression/activity), with symbols depicting gain-of-function and loss-of-function events. Percentages refer to ABC-DLBCL overall. This constellation of genetic events is also frequently observed in primary central nervous system lymphoma, PCNSL, and Waldenström macroglobulinemia. Note that, in the CBM complex, PKCβ directly phosphorylates CARD11.

In this framework, constitutive activation of the NF-κB signaling pathway downstream of a chronic active (e.g., antigen-dependent) BCR represents a genetic and phenotypic hallmark of ABC-DLBCL (MCD and/or BN2), where both the canonical [Figure 3] and non-canonical pathway are hijacked by a variety of genetic alterations (see next section). However, recent studies suggest that a small proportion of GCB-DLBCL also displays evidence of dysregulated non-canonical or canonical NF-κB activity [85,86]. Of note, lesions targeting known negative regulators of proximal BCR signaling (e.g., *LYN*, *PTPN6*, and *PRKCD*, among others) can be found in all DLBCL genetic subtypes, suggesting a pervasive role of BCR signaling in lymphomagenesis, through different modes of activation (“chronic, active” vs. “tonic”, or antigen-independent) and distinct downstream signaling cascades (NF-κB vs. PI3K).

The putative normal counterpart of GCB-DLBCL is unquestionably a GC B cell [7] that is undergoing somatic hypermutation of its rearranged Ig genes [87] and depends on tonic BCR signaling for its survival. However, different from Burkitt lymphoma (BL), the transcriptional profile of GCB-DLBCL does not display significant similarities to that of dark zone B cells, but resembles more closely GC B cells residing in the light zone, or intermediate populations that are transitioning between the dark zone and the light zone [22,88]. Consistent with this notion, BL and GCB-DLBCL feature distinct genetic make-ups, indicating the involvement of separate oncogenic pathways.

ABC-DLBCL, and in particular the MCD/C5 genetic subtype, has long been thought to derive from the transformation of partially differentiated antibody-secreting cells, or plasmablasts. Supporting this model are the elevated levels of IRF4 (a transcription factor upregulated upon commitment to plasma cell differentiation) and the low expression of genes like CD10 and BCL6, indicating the termination of the GC program [7,89]. The detection of somatically mutated rearranged Ig genes in ABC-DLBCL cases further documented that the COO of these tumours has experienced the GC reaction [87]. However, this cell is prevented from undergoing terminal differentiation in part due to inactivating mutations of BLIMP1, a further crucial plasma cell master regulator [90,91]. More recently, investigations based on single cell transcriptomics and mass cytometry profiling have pointed toward a potential origin from re-activated GC-derived memory B cells for at least a fraction of ABC-DLBCL cases [88,92]. Moreover, a mouse model recapitulating the loss of function of *TBL1XR1*, a genetic alteration commonly associated with MCD/C5 DLBCL, demonstrated a skewing in the differentiation of GC B cells to memory B cells that are preferentially engaged in secondary GC reactions [93]. These findings led to the hypothesis that iterative exposure to GC-associated DNA remodeling events would make these cells prone to accumulate additional genetic alterations that may ultimately lead to clonal expansion (see Ref. [92] for a critical review discussing this model). However, more recent in vivo studies based on the combination of four MCD-DLBCL-associated hallmark genetic aberrations (*Myd88L265P*, *Cd79B*, and *Prdm1* mutations, with or without *BCL2* amplification) revealed a different phenotype from that of *Tbl1xr1*-deficient mice, questioning a memory B-cell origin for this DLBCL molecular subtype [94]. In particular, the massive accumulation of dark zone GC B cells, greatly exceeding the number of memory B cells, and the monoclonality of GC B cells but not of memory B cells from tumor-bearing mice led to support a model in which mutated GC B cells expressing auto-reactive BCRs are preferentially recruited to spontaneous splenic GCs and represent the likely cell of origin of MCD-DLBCL [94]. N1 DLBCL, another subtype of ABC-DLBCL, may arise through the progressive malignant transformation of post-GC memory B cells, as their transcriptional profile is enriched in the memory B-cell signature [95]. In this regard, persistent antigen exposure, in the context of (1) self-reactivity or (2) chronic/recurrent infections, could account for both chronic BCR signaling activation in established tumours, and memory B-cell reactivation during lymphomagenesis.

### 4.2. Mutations Leading to the Activation of the Canonical NF-κB Pathway

Constitutive activation of the NF-κB signaling pathway is a distinctive feature of virtually all ABC-DLBCLs, originally revealed by the significant enrichment of NF-κB target genes [8] in the gene expression profiles of these lymphoma cases and by the nuclear localization of NF-κB transcription factors (RELA and p50, although p52 can also be found) in the tumour cells [11]. A genetic explanation for this phenotype was provided by structural and functional genomics, with the identification of recurrent oncogenic aberrations affecting proximal and distal members of the BCR and TLR pathway in a large proportion of cases, the highest prevalence being observed in the MCD and BN2 genetic subtypes. These genetic lesions maintain a chronic form of active BCR signaling, which is initiated through engagement by self-antigens [96]. The dependence of ABC-DLBCL from the BCR signaling pathway is underscored by the preferential sensitivity of these tumours to pharmacological agents that inhibit Bruton tyrosine kinase (BTK), the molecule linking the BCR to the NF-κB pathway, even in the absence of mutations targeting the BCR subunits CD79A/B [97].

In ~20% of ABC-DLBCL (50% of MCD/C5-DLBCL), gain-of-function mutations in the gene encoding CD79B target the protein immunoreceptor tyrosine-based activation motifs (ITAMs), thus abrogating a negative feedback loop mediated by the Lyn kinase [98]. Additionally, CD79B mutations decrease endocytic recycling of the BCR [98,99,100]. These events favor the microclustering of BCRs on the plasma membrane in a manner analogous to what happens upon engagement of this receptor by the antigen (in this case, a self-antigen), thereby augmenting BCR signaling [98]. Interestingly, while *CD79B* mutations are confined to MCD, BN2, and A53-DLBCL, mutations in the CD79A subunit are enriched in EZB-DLBCL, which is insensitive to NF-κB inhibition [80,101], suggesting qualitatively distinct roles of *CD79A* and *CD79B* mutations in lymphomagenesis.

Thirty percent of ABC-DLBCLs (>50% of MCD/C5-DLBCL) carry mutations of *MYD88,* among which a hotspot L265P substitution within the protein TIR (Toll/IL1 receptor) domain is selectively enriched in MCD-DLBCL [15,81]. *MYD88* encodes an adaptor molecule mediating activation of NF-κB as well as type I interferon responses downstream the TLR signaling pathway. The MYD88^L265P^ substitution was shown to induce the spontaneous assembly and activation of a protein complex containing the kinases IRAK1 and IRAK4, ultimately leading to engagement of the NF-κB signaling pathway [15]. Additionally, MYD88^L265P^ proteins cooperate with *CD79B* mutations to coordinately activate IKK and downstream NF-κB signaling through a protein supercomplex formed by MYD88, TLR9 at the surface of endolysosomes, and the BCR (named My-T-BCR) [101]. The discovery of this functional interaction provided a molecular explanation as to why BTK inhibition is exquisitely toxic to tumours carrying concurrent *MYD88* and *CD79B* mutations, which are only observed in MCD-DLBCL, while *MYD88^L265P^*-only mutant DLBCLs are insensitive to this treatment. The significance of other *MYD88* mutations, found in both ABC (non MCD) and GCB-DLBCL, remains to be established.

Constituents of the BCR “signalosome”, which relays signals from the BCR to the NF-κB signaling pathway, are also recurrent targets of somatic mutations in MCD and BN2 tumours. These include primarily *CARD11*, affected by point mutations in ~9% of ABC-DLBCL, and >15% of MCD-DLBCL cases, but also *BCL10* (mostly copy number gains in 5-10% of cases, preferentially in BN2/C1-DLBCL) and *MALT1* (<5% of cases). *CARD11* mutations typically introduce amino-acid substitutions in the coiled-coil domain, facilitating the formation of cytosolic aggregates and the spontaneous recruitment of downstream effector molecules, ultimately enhancing the ability of this signal adaptor molecule to transactivate NF-κB target genes [14].

*KLHL14* is another frequent target of mutations that, when inactivated, contributes to maintaining active BCR and NF-κB signaling in MCD-DLBCL. This protein functions as a CRL ubiquitin ligase and was shown to promote the turnover of immature glycoforms of the BCR subunits in the endoplasmic reticulum –which is different from mature BCRs on the plasma membrane– thus reducing total BCR levels [102]. In contrast, its loss by truncating mutations facilitated the assembly of the My-T-BCR supercomplex and promoted reduced sensitivity to ibrutinib [102].

Finally, the *TNFAIP3* gene is inactivated in ~30% of ABC-DLBCL and particularly in BN2 tumours, which also display mutations in its binding partner TNIP [11,12]. *TNFAIP3* encodes a dual function ubiquitin-modification enzyme (also known as A20) involved in the termination of NF-κB responses triggered by TLR and BCR stimulation. A20 removes K63-linked regulatory ubiquitins from a number of substrates via its OTU domain, and subsequently conjugates K48-linked ubiquitins via its zinc finger domains, targeting these proteins for proteasomal degradation. Thus, loss of A20 may cause inappropriately prolonged NF-κB responses, as indeed observed in *TNFAIP3* knock-out mice [103,104], and ultimately favor neoplastic transformation by enforcing pro-proliferative and anti-apoptotic mechanisms [11,12]. The tumour suppressor role of A20 is supported by the observation that enforced expression of a wild-type protein in *TNFAIP3*-null DLBCL cell lines results in cytoplasmic re-localization of the NF-κB complex and suppression of its activity, leading to apoptosis [11].

A variety of other genes encoding for NF-κB positive and negative regulators have been found mutated at lower frequencies in ABC-DLBCL. While space limits preclude an extensive description of these alterations, mutations at the following genes have been shown to portend clear functional consequences in facilitating the activation of the pathway: (i) *IKBKB*, preferentially found in N1-DLBCL (e.g., the gain of function V203I variant) [105]; (ii) *NFKBIA*, disrupted by somatic mutations in ST2-DLBCL and PMBL [81,106]; (iii) *NFKBIE*, which encodes the IκBε inhibitor and is also enriched in ST2-DLBCL; and (iv) *NFKBIZ*, a molecule with activating as well as inhibitory functions, the expression of which is increased in cases carrying mutations of its 3′-untranslated region (3′UTR) [107]. *NFKBIZ* was originally shown to contribute to ABC-DLBCL pathogenesis by upregulating specific NF-κB target genes [108]. *NFKBIZ* mutations tend to cluster with BN2 -DLBCL, which also displays *BCL10*, *PRKCB*, and *TRAF6* mutations, in addition to *TNFAIP3* and *TNIP1*, potentially promoting the formation or function of the CBM protein complex. If should be mentioned that, given the recent identification of a pervasive hypermutation activity targeting super-enhancer networks in DLBCL [109], it is expected that additional genes (or additional cases) will be found dysregulated in these tumours to sustain the aberrant activity of the NF-κB signaling cascade.

Different from ABC-DLBCL, GCB-DLBCL is characterized by a diverse form of BCR signaling activation, which mimics the tonic BCR engagement required for normal B-cell survival, and is NF-κB independent [110]. Consistently, GCB-DLBCL cells require the BCR subunits CD79A and CD79B for their survival but are insensitive to inhibition of signaling proteins that relay the signal to NF-κB [97,101]. The lack of NF-κB activity in most GCB-DLBCL seemingly contrasts with the frequent occurrence of *REL* gene amplifications [111], which has long been recognized as a defining genetic feature of this molecular subtype (15–30% of cases, vs. 5% of ABC DLBCLs) [112]. Indeed, earlier studies failed to obtain evidence of c-REL nuclear translocation in these tumours [111]. However, recent work reported some c-REL-DNA binding activity by EMSA in a subset of cases belonging to the GCB-subtype that also showed increased c-REL mRNA levels [85]. While it is possible that other genes in the amplicon represent the target of this genetic alteration, an alternative explanation for the frequent occurrence of *REL* amplifications in GCB-DLBCL tumour cells is that these lesions had been selected at an earlier stage of B-cell differentiation during the evolution of the “tumour-precursor cell” to a *bona fide* lymphoma. In this context, *REL* amplification and the consequent expression of higher amounts of c-REL may enhance the response of the precursor cell to microenvironmental signals that activate NF-κB, favoring the expansion of GC B cells, as observed in a c-REL transgenic mouse model [60]; this, in turn, may deregulate glycolytic metabolism and cell growth, which has been described as a critical c-REL activity in normal GC B cells [53].

### 4.3. Genetic Lesions Leading to Activation of the Non-Canonical NF-κB Pathway

Although less commonly, evidence for the involvement of the non-canonical NF-κB signaling cascade has been reported in both GCB and ABC-DLBCL. In a small subset of DLBCL samples, p52 can be detected in the nucleus [11], and truncating mutations/deletions of the *TRAF3* gene, often coexisting with *BCL6* translocations, are found in about 15% of cases, regardless of molecular subtype [113]. As a negative regulator of the non-canonical NF-κB pathway, biallelic *TRAF3* loss promotes the inappropriate stabilization of NIK, leading to IKK activation. Accordingly, enforced expression of NIK in the GC by conditional in vivo mutagenesis coupled with BCL6 deregulation caused GC hyperplasia with blockade of terminal differentiation and ultimately premature death in 100% of the animals, which developed IRF4-positive DLBCL [113]. Of note, single *Nik^stopFL^*Cγ1-Cre mice display overt plasma cell hyperplasia and do not succumb to tumours, suggesting that the oncogenic function of the non-canonical NF-κB pathway requires the concomitant disruption of terminal B-cell differentiation, achieved in the *Nik^stopFL^*Cγ1-Cre;IµHABCL6 model by deregulated *BCL6* expression. This synergistic interaction would be analogous to that observed between Blimp1 loss and a constitutively active Ikk2 in the compound *Blimp1*^fl/fl^*R26Stop*^FL^*Ikk2ca*Cγ1-Cre model, which develops a similar phenotype [114]. Mutations of *TRAF2* and *BIRC2/3* can also be found in a small subset of DLBCL, with *TRAF2* mutations being more common in GCB-DLBCL and *BIRC2/3* mutations preferentially associating with ABC-DLBCL.

A more recent study reported a previously unrecognized function for the alternative NF-κB subunit RELB in as many as 60% of DLBCL samples across the EZB and MCD subtype [86,115]. These tumours displayed RELB DNA-binding activity, as determined by EMSA, and a distinct gene expression profile enriched in genes linked to cell death and survival, metabolism, immune cell trafficking, inflammation, and proliferation. However, the RELB gene-expression signature did not correlate with nuclear p52 nor with classical NF-κB gene expression features. In line with these findings, RELB-positive DLBCLs were devoid of *MYD88^L265P^*, *CD79B*, and *CARD11* mutations, suggesting the involvement of a different circuit. Instead, these tumours are characterized by a significant correlation with *TRAF3* deletions and frameshift mutations. Genetic alterations in the *ITPKB* gene, encoding a regulator of B-cell survival in response to antigen presentation, and in *B2M*, encoding for an invariant subunit of the MHC-class I complex, were also significantly enriched in cases with RELB DNA-binding activity. Of note, RELB-EMSA positive cases were associated with inferior survival in this study, which could be sustained in part by the upregulated cIAP2/BIRC3 mRNA levels observed in response to DNA damaging agents. Additional studies in larger patient cohorts will be required to corroborate this model and potentially provide a framework for the development of RELB-targeted treatments to overcome chemoresistance in DLBCL.

### 4.4. Potential Role of Downstream NF-κB Transcription Factors in DLBCL Lymphomagenesis

The finding that NF-κB c-REL and the RELB/p52 heterodimer are independently required for the re-entry of light zone B cells into the dark zone, i.e., the putative normal cellular counterparts of GCB-DLBCL, suggests that those transcription factors may play a role in GCB-DLBCL pathogenesis [Figure 2]. Conversely, NF-κB RELA is dispensable at this developmental stage while it is required for plasmablastic development, which is associated with the ABC-DLBCL cell-of-origin. Thus, it will be interesting to determine the extent to which oncogenic NF-κB activation in ABC-DLBCL is mediated by a RELA-controlled biological program. For a discussion on the possible oncogenic activities of individual NF-κB transcription factors in lymphomagenesis, we refer the reader to Ref. [116]. The identification of the active, downstream NF-κB transcription factors co-opted in DLBCL subtypes and the elucidation of their transcriptional programs could guide the development of drugs that specifically inhibit oncogenic NF-κB target genes or pathways, with more limited toxicity.

## 5. Hodgkin Lymphoma

cHL is thought to originate from the malignant transformation of pre-apoptotic centrocytes, or light zone B cells, expressing the CD30 cell surface antigen [1]. The occurrence of somatic mutations that ‘cripple’ the antibody structure in the rearranged Ig variable region genes of cHL cells indicates that GC B cells that would be normally counter-selected due to their inability to bind antigen escape deletion by apoptosis [117].

The tumour cells of cHL, Hodgkin Reed Sternberg (HRS) cells, are characterized by the constitutive activation of both the canonical and non-canonical NF-κB signaling pathways [1]. In EBV-positive cHL cases (comprising ~30–40% of all diagnoses) NF-κB is activated through the viral genome-encoded LMP1 and LMP2a gene products that mimic the normal cellular CD40 and BCR signaling, respectively. Conversely, multiple genetic alterations have been found to disrupt regulators of the NF-κB signaling cascade, specifically in EBV-negative cHL.

The most frequent genetic lesions target the negative regulators of the canonical NF-κB pathway *TNFAIP3* and *NFKBIA* (encoding A20 and IĸBα) in 40% or 20% of cHL cases, respectively [4,5,6,12,17]. These mutations almost never occur in EBV-positive cases, indicating that EBV infection can substitute for some of the NF-κB-activating mutations in HRS cells. Somatic point mutations and deletions were also found in the NF-κB inhibitors *CYLD*, *NFKBIE* (IĸBε) and *TRAF3* [118,119,120]. In addition, copy number gains or amplifications target the genes encoding for the canonical NF-κB subunit c-REL and the non-canonical NF-κB pathway kinase NIK in 50% and 25% of cHL cases, respectively [121,122,123,124]. In contrast to GCB-DLBCL, *REL* locus-amplified cHL cases show nuclear c-REL translocation in HRS cells, suggesting a pathogenic role for c-REL in cHL. With 20% occurrence, *BCL3* is an additional gene of the NF-κB pathway that is affected by somatic copy number gains and rare translocations in cHL [1,125].

The rarity of the HRS cells in cHL tumours has complicated the determination of the pattern of NF-κB pathway component mutations within a single case. However, the observation that a number of HL cell lines comprise two or more genetic aberrations in NF-κB components have led to the suggestion that mutations in several NF-κB regulators are required for achieving strong constitutive activity of the pathway in HRS cells [1]. Finally, it should be noted that HRS cells are subjected to a multitude of strong NF-κB-activating signals from the tumour microenvironment that support their growth and survival, including activation through CD40, which induces both the canonical and non-canonical NF-κB pathways. Thus, the discernably strong NF-κB activity in HRS cells is likely to be the result of microenvironmental stimuli in conjunction with genetic aberrations in NF-κB pathway components, or the NF-κB-inducing action of EBV encoded genes in EBV^+^ cHL cases. The mutation-induced functional ablation of negative NF-κB regulators such as IĸBα and A20 presumably exacerbates the signals transmitted through cell surface receptors that activate the canonical NF-κB pathway. Likewise, elevated amounts of c-REL and NIK in the HRS cells due to gains and amplifications may enhance incoming signals from cell surface receptors activated by immune cells in the tumour microenvironment. Interestingly, in HL cell lines with constitutive NF-κB activity, NIK plays a central role in the activation of both the non-canonical and canonical NF-κB pathway, as demonstrated by the NIK-dependent processing of p100 as well as p105 [126]. This study also showed that canonical and non-canonical NF-κB heterodimers regulate both overlapping and distinct gene sets [126].

The assignment of HRS cells to a specific normal cellular counterpart is complicated by the fact that these cells, which belong to the B-lineage, lack the B-cell typical transcriptional program. Nevertheless, a recent comparative gene expression study could identify CD30^+^ B cells in the GC light zone as the presumptive counterparts of HRS cells [127]. In particular the strong c-MYC expression identified those cells as light zone B cells that are instructed by Tfh cells to recycle into the dark zone for further rounds of somatic hypermutation and clonal expansion. Similarly, the activity of the canonical NF-κB pathway, mediated by c-REL, and –in a mutually exclusive fashion– the non-canonical pathway was found to be a requirement for the recirculation of antigen-selected light zone B cells to the dark zone. On this basis, it may be suggested that a light zone B cell expressing a BCR with insufficient affinity or crippling mutations and thus normally destined to die by neglect, escapes apoptosis as the result of genetic mutations leading to constitutive NF-κB activity [Figure 2]. In concert with other mutations, this cell eventually transforms into a *bone fide* HRS cell. The constitutive activities of c-REL and RELB/p52 in such pre-apoptotic light zone B cells may contribute to lymphomagenesis by promoting cellular metabolism and proliferation, two biological functions that have been associated with these NF-κB subunits in light zone B Cells [48,53].

## 6. Primary Mediastinal Large B-Cell Lymphoma

PMBL is thought to originate from the malignant transformation of GC or post-GC B cells, possibly medullary thymic B cells based on the location of the tumours within the body. PMBL is a non-Hodgkin lymphoma; however, the tumour cells show significant similarities with cHL with regard to both their gene expression and the pattern of genetic aberrations [128]. Moreover, constitutive activation of the NF-κB pathway has been noted as a defining feature of PMBL, evidenced by an NF-κB transcriptional signature and by the nuclear translocation of NF-κB transcription factor complexes in the tumour cells.

The most frequent mutations affecting NF-κB pathway components are *REL* amplifications, *TNFAIP3* deletions and *NFKBIE* alterations, occurring in 50%, 30% and 23% of cases, respectively [17,119,129,130,131,132]. While these genetic aberrations are also found in cHL, PMBL is further characterized by amplifications of genomic loci that comprise the *BCL10* and *MALT1* genes, which act in the BCR signaling pathway [131]. A recent whole-exome sequencing analysis has identified additional genes encoding NF-κB pathway components that are recurrently mutated in PMBL, namely *IKBKB* (encoding IKKβ), *NFKBIA* (IĸBα), *TRAF3* and *RELB* [133]. Overall, these mutations lead to the constitutive activation of both the canonical and non-canonical pathways.

As mentioned, the pattern of NF-κB pathway mutations in PMBL is similar to that observed in cHL and largely different from the one characterizing ABC-DLBCL, in that activating mutations of upstream BCR pathway molecules (such as *CD79* and *CARD11*) or TLR pathway components (*MYD88*) do not occur. The absence of such events in PMBL may suggest either that BCR and/or TLR stimulation do not play a tumour-promoting role in this disease, or that mutations in inhibitory NF-κB factors are sufficient to induce a stronger NF-κB response to stimuli from the tumour microenvironment and/or to soluble factors.

While the exact normal cellular counterpart of PMBL is still unknown, the similarities with cHL suggest that the PMBL precursor represents a B cell—possibly located in the light zone of the GC—that is subjected to receptor-mediated NF-κB signaling via both the canonical and non-canonical pathways [Figure 2]. The observed frequent amplification of the *REL* locus may further point towards a developmental stage of the PMBL precursor cell that is under the control of a c-REL-mediated transcriptional program, which exerts crucial functions at the B-cell activation and GC dark zone–light zone recirculation steps. Similarly, the occurrence of *TRAF3* and *RELB* mutations may indicate a biological role for the non-canonical NF-κB pathway in PMBL pathogenesis. However, in contrast to cHL, amplification of the *MAP4K14* locus encoding the central activator of the non-canonical pathway, NIK, does not appear to be a genomic feature of PMBL.

## 7. Therapeutic Implications

The frequent oncogenic activation of NF-κB in lymphomas identifies this signaling pathway as a potential critical target for pharmacological intervention [134]. As such, several drugs that at least in part act through inhibition of NF-κB (directly or indirectly) have been proposed over the years to treat B-cell malignancies showing dependency on this signaling cascade. However, two main issues have limited their usability and efficacy: lack of specificity and systemic toxicity [135] (an exhaustive review on the topic, including combination therapies, can be found in Ref. [110]).

Although their activity is not restricted to NF-κB, proteasome inhibitors leverage the unfolded protein response co-opted by the tumour cells to cope with the increased build-up of proteins, and were postulated to impair the degradation of the canonical NF-κB inhibitor IκBα, thereby preventing activation of this pathway [136]. Novel agents developed to treat plasma cell disorders, like bortezomib and carfilzomib, have shown remarkable preclinical and clinical antitumour activity, and were approved by the FDA for the treatment of both relapse/refractory and newly diagnosed MM [137,138]. These compounds have demonstrated some favorable effect in clinical trials for the treatment of non-GCB DLBCL [139,140]. However, differences did not reach significance, and evidence obtained in later studies suggested that bortezomib induces activation of the canonical pathway [141,142]. Moreover, both agents cause side effects that limit their applicability for treatment, probably reflecting the wide-ranging consequences associated with inhibiting a central cellular pathway such as proteasomal degradation.

A more powerful approach for the treatment of tumours addicted to chronic active BCR signaling caused by genetic mutations upstream of BTK, such as those targeting the My-T-BCR complex (e.g., ABC-DLBCL of the MCD type, primary central nervous system lymphoma (PCNSL), and Waldenström Macroglobulinemia (WM)) emerged from the development of the small molecule ibrutinib. This BTK covalent inhibitor binds to a cysteine adjacent to the BTK active site, abolishing the activity of the canonical NF-κB pathway [143]. Ibrutinib showed selective toxicity to ABC, but not GCB-DLBCL cell lines in preclinical studies and early clinical trials [97], and exceptional responses were reported among young patients in a recent phase III clinical study of ibrutinib plus R-CHOP [144]. Although resistance almost inevitably occurs, due to genetic mechanisms (mutations in *BTK* [145] or *PLCG2*) or to acquired non-genetic rewiring of the BCR signaling cascade (i.e., PLCG2 activation by RAC2 bypassing BTK) [146], clinically available drugs were recently identified in a CRISPR-screen that can counteract “epigenetic” ibrutinib resistance in cell lines, suggesting alternative combinatorial approaches. A second-generation BTK inhibitor, acalabrutinib, demonstrated superior specificity, as it does not affect ITK and other kinases that present cysteines at homologous positions [147], and although its toxicity profile is similar, side effects are observed at possibly lower frequencies [148].

Given the dependency of ABC-DLBCL on the CARD11-BCL10-MALT1 complex, a growing interest has been seen in the development of small molecules targeting MALT1. These include allosteric MALT1 protease inhibitors, currently in phase I clinical trials, and compounds that block the scaffolding function of MALT1 [149]. These drugs were shown to overcome resistance to BTK inhibitors in preclinical models, supporting MALT1 as a promising target for ABC-DLBCL.

In the past, much attention has focused on the drug-mediated inhibition of IKKβ, which in cell culture worked very effectively [150]. However, IKKβ inhibitors exhibited marked toxicity in clinical trials, essentially halting their potential therapeutic use. Additional drugs under development that target NF-κB pathway components upstream of the IKK complex are TLR antagonists and IRAK4 inhibitors, which are potentially interesting for the treatment of DLBCL and WM with *MYD88* mutations. The central regulator of the non-canonical pathway NIK represents a further attractive candidate for NF-κB inhibition, but NIK inhibitors have not made it to clinical trials.

Until now, most efforts concentrated on inhibiting upstream components of the NF-κB pathway. A strategy to inhibit aberrant NF-κB activation in a more specific fashion, and thus potentially reducing systemic toxicity, would be to target the downstream mediators of the pathway, including the individual NF-κB transcription factors or select target genes that are oncogenic in lymphomas. An example for the successful pharmacologic targeting of an NF-κB target gene by specific inhibitors is the GADD45β/MKK7 complex, which was demonstrated in a MM xenograft model [151]. It is becoming increasingly clear that the separate NF-κB subunits can have distinct roles in cancer cells contributing to tumorigenesis [152]; hence, specific inhibition of individual NF-κB subunits could be exploited for therapies against aberrant NF-κB activity with fewer side effects. As an example, a small molecule c-REL inhibitor showed activity against human lymphoma cells in a xenograft model (although, at least in mice, the drug also targets the RELA subunit) [153]. Moreover, this drug inhibited c-REL function in T regulatory cells in an animal model of melanoma, thus delaying tumour growth [154].

By leveraging the ubiquitin-proteasome pathway, proteolysis-targeting chimeras (PROTACs) have the power to selectively degrade intracellular proteins that are otherwise difficult to target with small molecule inhibitors or are ‘undruggable’ [155]. Recently, BTK was reported to be degraded via a PROTAC approach in ibrutinib-sensitive and resistant lymphoma cell lines [156]. These results provide a proof-of-principle for the use of the PROTAC technology in the treatment of cases where resistance to small molecule inhibitors developed due to mutations. Improvements in strategies aimed at more effectively targeting mutated intracellular oncoproteins or oncogenic transcription factors for proteasomal degradation may spur the development of PROTACs that ablate the function of specific NF-κB subunits. As elaborated in the preceding sections, the distinct biological roles that individual NF-κB transcription factors play in different GC B-cell subsets may favor cell growth or survival of malignancies originating from those cell stages, and make these subunits conceivable targets for proteasomal degradation. For example, c-REL may be a suitable target for GC tumours with strong NF-κB pathway activity, RELA for plasmablast-associated tumours, and RELB and/or NF-κB2 for the subset of DLBCL cases with nuclear translocation of these subunits. A more selective approach for inhibiting aberrant NF-κB signaling through targeting the downstream transcriptional mediators may result in a substantial reduction in systemic toxicity.

## 8. Conclusions

Constitutive activation of the NF-κB pathway through genetic aberrations and signals from the tumour microenvironment plays a major oncogenic role in GC-derived lymphomas, identifying inhibition of NF-κB signaling as a conceivable strategy for lymphoma treatment. The comprehensive identification of NF-κB mutations in distinct lymphoma subtypes together with new insights from the mechanistic dissection of NF-κB pathway activation in the normal cellular counterparts of these tumours, i.e., GC and post-GC B cells, are beginning to establish a framework that may be exploited for the development of effective, more specific NF-κB inhibitors lacking systemic side effects.
